# Association of anaesthesia type with one-year mortality after surgery in elderly patients: a secondary retrospective cohort study

**DOI:** 10.1186/s12871-025-03191-y

**Published:** 2025-07-01

**Authors:** Ping Jin, Fengjiao Lu, Rongzhi Zhang, Panpan Lü, Shixiong Gao

**Affiliations:** 1 Department of Anesthesiology and Surgery, The Second Hospital & Clinical Medical School, Lanzhou University, Lanzhou, Gansu 730030 China; 2 Department of Anesthesiology, The First People’s Hospital of Tianshui, Tianshui, Gansu 741000 China

**Keywords:** Regional anaesthesia, General anaesthesia, One-year mortality, Elderly population, Retrospective cohort study

## Abstract

**Objective:**

To examine whether regional anaesthesia (RA) versus general anaesthesia (GA) is associated with the one-year postoperative mortality among the older surgical patients.

**Methods:**

We conducted a single-center retrospective cohort study from 2012 to 2016. Patients aged 70 years or older who underwent surgery were included, and those who underwent transplantation, burn surgery, or minor procedures were excluded. The primary exposure was anaesthesia type (RA vs. GA); the main outcome was one-year all-cause mortality, which was verified through hospital records and a national registry. Demographic, clinical, and laboratory variables were included as covariates. Multivariable-adjusted logistic regression models were used to evaluate the independent effect of anaesthesia methods on one-year mortality. Kaplan-Meier curves assessed survival rates by anaesthesia method, with log-rank tests comparing the curves.

**Main results:**

Among 16,599 older adults, 29.7% received RA. The one-year mortality rate was lower in the RA group (6.44%) than in the GA group (9.52%), yielding an adjusted odds ratio of 0.72 (95% CI, 0.63–0.82). K‒M analyses revealed improved survival in the RA group (log-rank *P* < 0.05). Propensity score matching and inverse probability weighting analyses corroborated these findings. The E-value of 2.12 demonstrates the robustness of the results against unmeasured confounding.

**Conclusions:**

Regional anaesthesia may be linked to better one-year survival in older patients. Although other confounding factors cannot be excluded, these findings underscore the need for multicenter, prospective investigations to inform perioperative decisions in geriatric populations.

**Supplementary Information:**

The online version contains supplementary material available at 10.1186/s12871-025-03191-y.

## Introduction

Globally, nations are observing a significant increase in both the absolute number and relative proportion of elderly individuals within their populations [1]. There is a corresponding surge in surgical demand [2]. Older patients face increasing perioperative complications and mortality with advancing age [3, 4]. The anaesthesia technique represents a potentially modifiable risk factor, prompting extensive research into its relationship with patient outcomes [5]. Although numerous studies have examined the association between the anaesthesia approach and mortality, the findings remain inconsistent or even contradictory [6–9]. Many existing investigations focus on lower-limb or hip procedures, with most randomized evidence drawn from hip fracture surgery [5, 10] or comparisons of regional anaesthesia with general anaesthesia in hip fracture cohorts [11], limiting the scope of evidence. Different surgical techniques and procedures can greatly influence recovery and outcomes. Moreover, many studies rely on 30-day mortality as the main endpoint [12], which may not adequately capture the long-term impact of anaesthesia on survival [13]. Older patients often have multiple comorbidities and complexities, and the 30-day mortality rate may not capture the delayed risks and complications. Therefore, the one-year mortality rate provides a more comprehensive assessment. Research on GA versus RA continues to expand into areas such as cardiac and major vascular surgery [14, 15], neurosurgery [16], and carotid endarterectomy [17].

Consequently, our study aims to encompass diverse surgical types and longer-term outcomes. We hypothesize that, compared to GA, RA may be associated with lower one-year postoperative mortality in older patients, thereby providing more evidence to guide anaesthesia decision-making in older patients.

## Methods

### Data sources and participants

This study is a retrospective analysis of a comprehensive vertical cohort established by Yilin Eileen Sim and her colleagues at Singapore General Hospital, one of the best hospitals in Singapore and the largest and oldest public hospital in the country. The dataset utilized is available in the Dryad repository at https://datadryad.org/dataset/doi:10.5061/dryad.5772v, generously provided by Yilin et al., who have relinquished all copyrights and associated ownership rights. Consequently, these data are permissible for secondary analysis without violating any intellectual property rights. The data were originally derived from the published work of Sim et al. [18].

The original dataset’s clinical records were sourced from the hospital’s Clinical Information System, specifically the Sunrise Clinical Manager (SCM) by Allscripts, IL, United States. Mortality and follow-up information from the original study were integrated with national electronic health records to enhance data comprehensiveness. The dataset encompasses the following variables: sex, age, race, history of cerebrovascular accidents (CVAs), history of ischemic heart disease (IHD), history of diabetes mellitus requiring insulin therapy (DMI), history of congestive heart failure (CHF), creatinine levels, surgical priority, surgical risk classification, American Society of Anesthesiologists physical status (ASA-PS), red cell distribution width (RDW), chronic kidney disease (CKD) stage, degree of anemia, type of anaesthesia administered (general or regional), duration of follow-up in days, and survival status [18, 19]. This database is a comprehensive perioperative database that focuses on various perioperative factors and the one-year postoperative survival of adult patients. In our secondary analysis, we strictly used the data from the database without making any modifications to the original data.

The original investigation received approval from the Institutional Review Board (SingHealth CIRB 2014/651/D), which also granted a waiver for individual informed consent [18]. As detailed in prior publications, the present study constitutes a secondary analysis of the aforementioned data and did not require additional ethical approval [20].

### Study sample

**Fig. 1:**
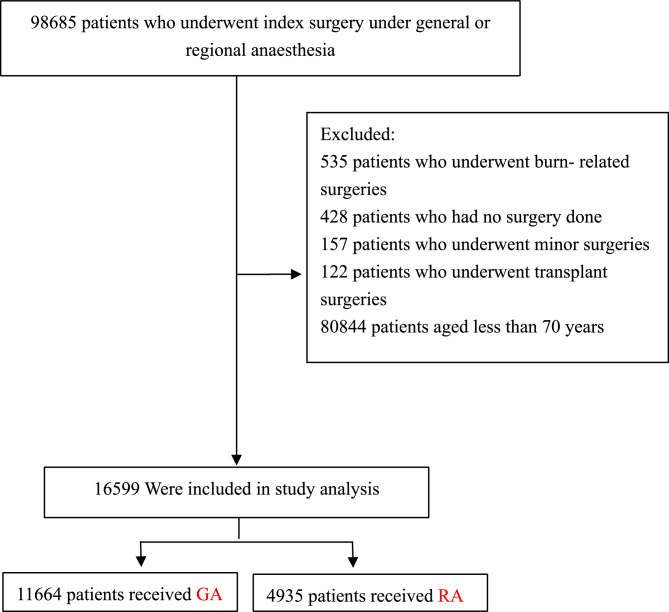
Flow chart of the study population

Consistent with the original study, patients aged 18 years and older who underwent surgery at Singapore General Hospital from January 1, 2012, to October 31, 2016, were included. The exclusion criteria were as follows: (1) transplantation or burn surgeries; (2) no surgical procedures; and (3) minor surgeries [18]. The initial cohort comprised 97,443 patients. For this analysis, 80,844 patients younger than 70 years were excluded, resulting in a final sample of 16,599 elderly patients. The patients selection process is illustrated in Fig. 1.

### Variable definitions

The primary exposure variable was the anaesthesia method administered during surgery, which was categorized as general or regional anaesthesia on the basis of detailed surgical records. These clinical records were sourced from the institution’s clinical information system (Sunrise Clinical Manager (SCM), Allscripts, IL, USA) and stored in our enterprise data repository and analytics system (SingHealth-IHiS Electronic Health Intelligence System—eHINTS). GA is a reversible loss of consciousness induced by intravenous or inhalational agents, typically requiring advanced airway management; RA blocks nerve conduction in a targeted region, allowing the patient to remain awake, often through neuraxial or nerve-block techniques [10, 18]. It is important to note that the database utilized for this study does not differentiate between combined techniques. Therefore, patients receiving combined GA and RA were classified and included under the GA group based on the structure of the database. The outcome variable was one-year mortality, defined as death from any cause within one year postsurgery, determined through hospital life records and the national death registry system, and recorded as a binary variable (alive or dead). Laboratory examinations, including serum creatinine, serum haemoglobin, and red cell distribution width (RDW) measurements, were conducted within 90 days of surgery. Anaemia was defined via the World Health Organization’s sex-specific classification: mild anaemia (haemoglobin 11.0–12.9 g/dL for males and 11.0–11.9 g/dL for females), moderate anaemia (8.0–10.9 g/dL), and severe anaemia (≤ 8.0 g/dL) [21]. RDW was reported as the coefficient of variation of red blood cell volume, categorized as high RDW (> 15.7%) or normal RDW (10.9–15.7%). During follow-up, 88.7% of participants were successfully monitored for one year, with a mean follow-up duration of 258 days [18].

### Missing data processing

Missing data accounted for 8.43% of the variables in the dataset analysed for this study, a common issue in observational studies. The lollipop chart (Supplementary Fig. 3) delineates missing rates across clinical variables. To mitigate bias from missing covariates that could compromise statistical efficiency, multiple imputation methods were employed [22, 23]. Dummy variables were used to indicate missing covariate values [24]. Analytical procedures were applied to both the original and imputed datasets for comprehensive comparison. Subsequently, regression analyses and propensity score matching were conducted on the multiple imputed datasets to ensure the robustness and reliability of the findings (Table 1).

**Table 1:** Associations between anaesthesia type and the endpoint of one-year mortality, as analysed in the crude analysis and multivariable analysis via logistic regression

Dataset	Analysis	NO.	One year Mortality
Original	No. of events/no. of patients at risk (%)		
	GA		1110/11,664(9.52)
	RA		318/4935(6.44)
Original	Multivariable analysis - Odds Ratio (95% CI) *P* value		
	Nonadjusted	16,599	0.65 (0.58, 0.75) < 0.001
	Adjust I	16,593	0.67 (0.59, 0.76) < 0.001
	Adjust II	10,323	0.72 (0.63, 0.82) < 0.001
DV-Imp	Multivariable analysis - Odds Ratio (95% CI) *P* value		
	Adjust II	16,599	0.73 (0.61, 0.88) < 0.001
MI	Multivariable analysis - Odds Ratio (95% CI) *P* value		
	Adjust II	16,599	0.72 (0.62, 0.83) < 0.001

The nonadjusted model was adjusted for none. The adjusted I model was as follows: race; gender. The adjusted II model was adjusted for race; gender; ASAPS; CVA; IHD; CHF; DMI; creatinine; surgery risk; anaemia; grade of kidney disease; priority of surgery; perioperative transfusion; MCV; RDW. DV-Imp: Dataset with missing values handled via dummy variable adjustments. MI: Multiple imputation datasets

### Statistical analysis

The participants were stratified by anaesthesia type. Continuous variables are presented as the means ± standard deviations, and categorical variables are presented as frequencies or percentages. Group differences were assessed via the chi-square test for categorical variables and one-way ANOVA for continuous variables (Table 2). Multivariable-adjusted logistic regression models were constructed, including Model 1 with no covariate adjustments, Model 2 adjusted for sociodemographic factors, and Model 3 further adjusted for the additional covariates listed in Table 2. Kaplan‒Meier curves were plotted and compared between anaesthesia methods via the log-rank test.

**Table 2:** Patient characteristics according to anaesthesia type

Variables	GA	RA	SD(CI%)	*P* value
N	11,664	4935		
Survival time	342.33 ± 77.49	350.55 ± 61.49	0.12 (0.08, 0.15)	< 0.001
Gender			0.12 (0.09, 0.15)	< 0.001
Female	5853 (50.18)	2770 (56.13)		
Male	5811 (49.82)	2165 (43.87)		
Race			0.11 (0.08, 0.14)	< 0.001
Chinese	9878 (84.69)	4291 (86.95)		
Malay	582 (4.99)	256 (5.19)		
Indian	542 (4.65)	221 (4.48)		
Others	657 (5.63)	166 (3.36)		
NA	5 (0.04)	1 (0.02)		
Grade of kidney disease			0.25 (0.22, 0.29)	< 0.001
G1	3444 (29.53)	1507 (30.54)		
G2	4832 (41.43)	2062 (41.78)		
G3	2075 (17.79)	892 (18.07)		
G4 and 5	691 (5.92)	417 (8.45)		
NA	622 (5.33)	57 (1.16)		
RDW			0.28 (0.25, 0.32)	< 0.001
≤ 15.7%	9611 (82.40)	4433 (89.83)		
> 15.7%	1363 (11.69)	446 (9.04)		
NA	690 (5.92)	56 (1.13)		
MCV			0.27 (0.24, 0.31)	< 0.001
Norma	9639 (82.64)	4389 (88.94)		
Low	979 (8.39)	336 (6.81)		
High	356 (3.05)	154 (3.12)		
NA	690 (5.92)	56 (1.13)		
Anemia			0.13 (0.09, 0.16)	< 0.001
None	6249 (53.58)	2708 (54.87)		
Mild	2748 (23.56)	1163 (23.57)		
Moderate	2311 (19.81)	1003 (20.32)		
Severe	90 (0.77)	16 (0.32)		
NA	266 (2.28)	45 (0.91)		
CVA			0.03 (−0.01, 0.06)	0.329
No	7664 (65.71)	3197 (64.78)		
Yes	605 (5.19)	246 (4.98)		
NA	3395 (29.11)	1492 (30.23)		
IHD			0.07 (0.04, 0.11)	< 0.001
No	6462 (55.40)	2805 (56.84)		
Yes	1782 (15.28)	627 (12.71)		
NA	3420 (29.32)	1503 (30.46)		
CHF			0.01 (−0.02, 0.05)	0.747
No	8271 (70.91)	3488 (70.68)		
Yes	387 (3.32)	155 (3.14)		
NA	3006 (25.77)	1292 (26.18)		
DMI			0.06 (0.02, 0.09)	0.002
No	8233 (70.58)	3412 (69.14)		
Yes	350 (3.00)	198 (4.01)		
NA	3081 (26.41)	1325 (26.85)		
Creatinine			0.08 (0.04, 0.11)	< 0.001
No	9394 (80.54)	3906 (79.15)		
Yes	485 (4.16)	289 (5.86)		
NA	1785 (15.30)	740 (14.99)		
Perioperative transfusion			0.21 (0.17, 0.24)	< 0.001
0 unit	10,207 (87.51)	4614 (93.50)		
1 unit	1166 (10.00)	261 (5.29)		
2 or more units	291 (2.49)	60 (1.22)		
Priority of surgery			0.03 (−0.00, 0.06)	0.078
Elective	9314 (79.85)	3881 (78.64)		
Emergency	2350 (20.15)	1054 (21.36)		
Surgery risk			0.58 (0.55, 0.62)	< 0.001
Low	3950 (33.86)	1750 (35.46)		
Moderate	5673 (48.64)	3108 (62.98)		
High	1036 (8.88)	77 (1.56)		
NA	1005 (8.62)	0 (0.00)		
ASAPS			0.23 (0.20, 0.27)	< 0.001
1	270 (2.31)	125 (2.53)		
2	6093 (52.24)	2965 (60.08)		
3	4250 (36.44)	1589 (32.20)		
4	574 (4.92)	76 (1.54)		
5&6	11 (0.09)	0 (0.00)		
NA	466 (4.00)	180 (3.65)		
One year mortality			0.11 (0.08, 0.15)	< 0.001
No	10,554 (90.48)	4617 (93.56)		
Yes	1110 (9.52)	318 (6.44)		

For continuous variables: *(N) Mean ± SD, SD* standardized differences, *CVA* history of previous cerebrovascular accidents, *IHD* history of ischemic heart disease, *DMI* history of diabetes mellitus on insulin, *CHF* congestive heart failure, *ASA-PS* American Society of Anesthesiologists Physical Status, *CKD* chronic kidney disease, *RDW* red cell distribution width

To examine the robustness of the results, we conducted sensitivity analyses. Missing data were handled via multiple imputation via random forests, generating five complete datasets to analyse the relationships between anaesthesia methods and one-year postoperative mortality. Each dataset underwent a multivariable-adjusted logistic regression model adjusting for all covariates. Propensity score matching (PSM) was performed by estimating propensity scores through logistic regression based on all covariates. This was followed by nearest neighbor matching using a caliper of 0.2 and a 1:1 ratio. One imputed dataset will be showcased in the results section to ensure clarity. Covariate balance before and after matching was assessed via standardized mean difference (SMD). The specific analyses included a multivariate-adjusted logistic regression model applied to the PS-matched cohort with all covariates [25–27], propensity score adjustment via a multivariable logistic regression model with the same strata and covariates while additionally adjusting for propensity scores [28], and inverse probability of treatment weighting (IPTW) [29], where weights were derived from propensity scores to balance covariates between groups, followed by a weighted multivariable logistic regression adjusting for all covariates to assess the impact of anaesthesia methods on postoperative mortality. Subgroup analyses were conducted by stratifying each imputed dataset on the basis of predefined criteria (e.g., race, sex, comorbidities), with logistic regression adjusting for all covariates except the stratification variable. The results from the five imputed datasets were combined via Rubin’s rules to obtain pooled effect estimates [24].

In addition, we explored the possibility of unmeasurable confounding effects between anaesthesia methods and one-year mortality after surgery by calculating E values [30]. All the results are reported according to the STROBE statement [31]. All the statistical analyses were performed via R software (http://www.R-project.org; The R Foundation) and Empower-Stats (https://www.empowerstats.net/cn/; X&Y Solutions, Inc., Boston, MA, United States). Two-sided *p* values < 0.05 were considered statistically significant.

## Results

A total of 16,664 elderly patients received GA and RA (Table 2). Compared with the GA group, the RA group presented a significantly longer mean survival time (350.55 ± 61.49 days vs. 342.33 ± 77.49 days, *P* < 0.001). The sex distribution differed significantly, with a greater proportion of females in the RA group (56.13%) than in the GA group (50.18%, *P* < 0.001). The majority of patients in both cohorts were Chinese, with a slightly greater prevalence in the RA group (86.95% vs. 84.69%, *P* < 0.001). Patients in the RA group presented more advanced chronic kidney disease (CKD) stages (*P* < 0.001) and greater red cell distribution widths (RDW > 15.7%) (11.69% vs. 9.04%, *P* < 0.001). Anaemia was more prevalent in the GA group across all severity levels (*P* < 0.001). Comorbidities such as ischemic heart disease (IHD) were more common in the GA cohort (15.28% vs. 12.71%, *P* < 0.001). Additionally, perioperative transfusion rates were significantly higher in the GA group (10.00% vs. 5.29%, *P* < 0.001).

### One-year mortality after surgery

The one-year mortality rate was significantly lower in the RA group than in the GA group (6.44% vs. 9.52%). Multivariate logistic regression analyses demonstrated that RA was associated with a reduced one-year mortality risk across different models and datasets. In the unadjusted model, RA was associated with a 35% decrease in mortality odds (OR = 0.65; 95% CI: 0.58–0.75). Adjusted Model I (race and sex): The odds ratio was 0.67 (95% CI: 0.59–0.76). Adjusted Model II (comprehensive clinical variables): The OR was 0.72 (95% CI: 0.63–0.82). When data were missing, adjusted Model II yielded an OR of 0.73 (95% CI: 0.61–0.88), and multiple imputation resulted in an OR of 0.72 (95% CI: 0.62–0.83). These consistent findings demonstrate that RA is independently associated with a significant reduction in one-year mortality compared with GA, even after adjusting for potential confounders and addressing missing data.

The nonadjusted model was adjusted for none. The adjusted I model was as follows: race; gender. The adjusted II model was adjusted for race; gender; ASAPS; CVA; IHD; CHF; DMI; creatinine; surgery risk; anaemia; grade of kidney disease; priority of surgery; perioperative transfusion; MCV; RDW. DV-Imp: Dataset with missing values handled via dummy variable adjustments. MI: Multiple imputation datasets.

**Fig. 2:**
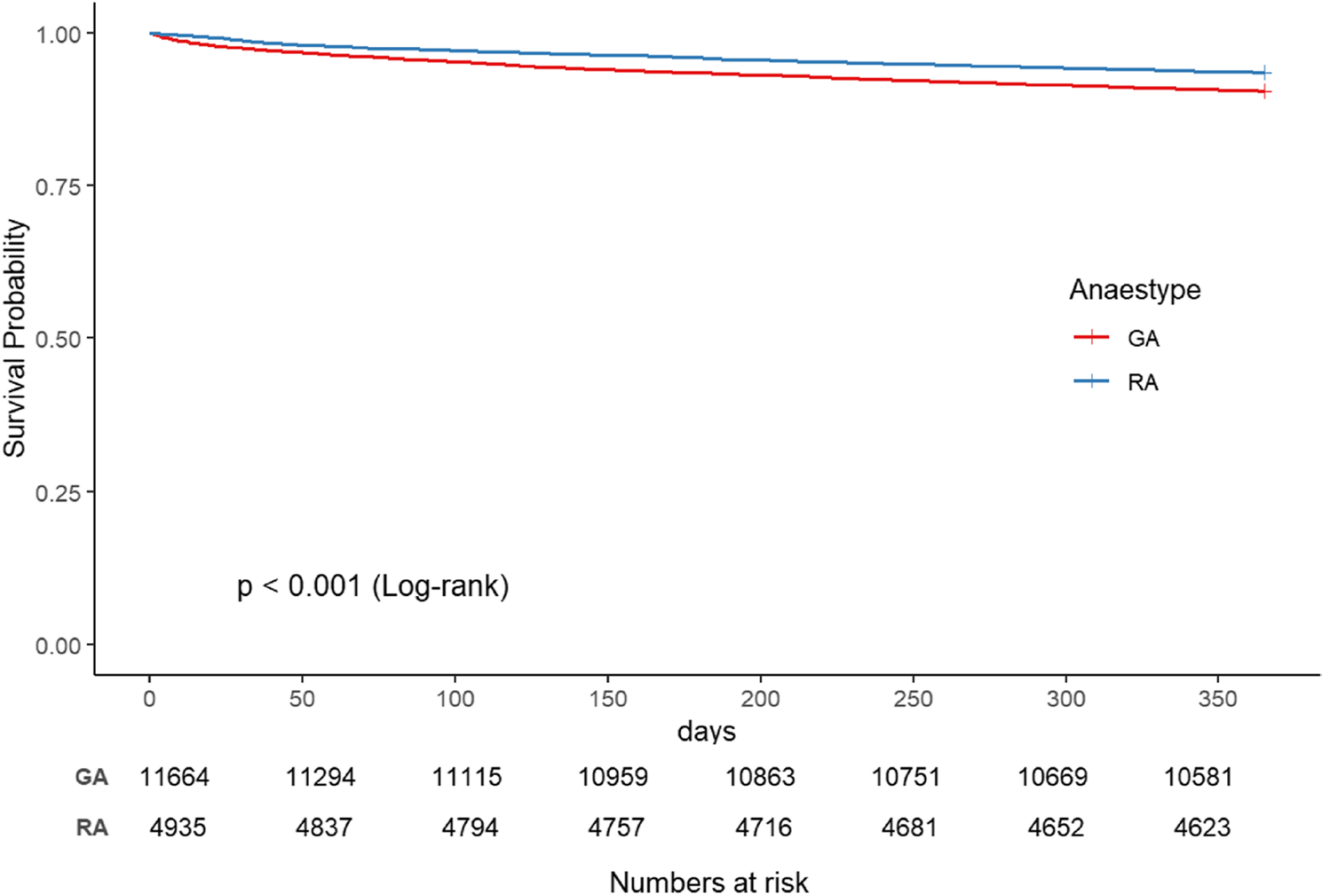
Kaplan–Meier survival curve based on anaesthesia type in the original cohort. Kaplan–Meier analysis of one-year mortality after surgery with GA and RA in the original cohort (log-rank, *P* < 0.001)

The survival curve for the RA group was slightly better than that for the GA group, indicating a relatively better survival probability for the GA group throughout the observation period. As time progresses, both groups’ survival curves tend to stabilize, reflecting a decline in survival probability. The log-rank test results revealed a statistically significant difference in survival between the GA and RA groups (*p* < 0.001), suggesting that the type of anaesthesia has an impact on patient survival (Fig. 2).

### Analysis results through different confounding control methods

To illustrate the information and effects before and after propensity score matching, we selected one dataset to present its baseline characteristics and matching outcomes (Supplementary Fig. 4). We conducted multivariable logistic regression analyses on five imputed datasets to evaluate the relationship between anaesthesia type and one-year mortality, calculating the Akaike information criterion (AIC) and Bayesian information criterion (BIC) for each dataset. Ultimately, we chose the fourth dataset for presentation, which had the lowest AIC (8003) and BIC (8219) [32]. Although we display the matching effects of the fourth dataset (Supplementary Table 3), the final matching analysis results are based on the combined effect estimates from all five datasets [24] (Supplementary Table 4). A total of 9,868 patients were matched (4,934 per group), representing 59.45% of the original sample. The maximum SMD was 0.051 for surgery priority, with most variables showing an SMD < 0.1, indicating high-quality matching and minimal bias between anaesthesia groups (Supplementary Table 3). Propensity score analyses consistently demonstrated the protective effect of RA on one-year postoperative mortality among elderly surgical patients. The propensity score method revealed an odds ratio (OR) of 0.76 (95% CI: 0.65–0.88; *p* = 0.001). Propensity score adjustment analyses revealed an OR of 0.74 (95% CI: 0.64–0.86; *p* < 0.001). Inverse probability of treatment weighting (IPTW) analyses further confirmed these findings, with an average treatment effect for all (ATE) of 0.71 (95% CI: 0.60–0.84; *p* < 0.001), an average treatment effect for treated (ATT) of 0.72 (95% CI: 0.62–0.83; *p* < 0.001), and an average treatment effect for the control (ATC) of 0.71 (95% CI: 0.59–0.86; *p* < 0.001) (Supplementary Table 4).

### Sensitivity analysis

Compared with GA, RA was associated with lower mortality rates across postoperative time intervals (30 days: OR, 0.73; 95% CI, 0.49–1.09; 60 days: OR, 0.76; 95% CI, 0.56–1.04; 90 days: OR, 0.74; 95% CI, 0.57–0.98; 180 days: OR, 0.68; 95% CI, 0.54–0.85; 360 days: OR, 0.73; 95% CI, 0.61–0.88) (Supplementary Table 5). To assess the robustness of our findings, we calculated an E-value of 2.12. This means an unmeasured confounder would need to be independently associated with both the exposure and outcome by at least 2.12 times to negate the observed association. Therefore, a factor not considered must significantly enhance the likelihood of both variables to challenge our results, indicating that our findings are relatively robust against potential unmeasured confounding.

In a matched cohort of 4,931 patients each in the GA and RA groups, one-year mortality rates were compared. In the Adjusted Model II (controlling for comprehensive clinical variables), the odds ratio (OR) for mortality with GA versus RA was 0.76 (95% CI: 0.65–0.88; *P* < 0.001).For low-risk surgical patients (*n* = 3,400), the Adjusted Model II (excluding surgery risk) showed an OR of 1.02 (95% CI: 0.78–1.33; *P* = 0.88). In the mid-to-high-risk group (*n* = 6,462), the OR was 0.54 (95% CI: 0.43–0.68; *P* < 0.0001). These results suggest that RA may provide additional benefits compared to GA for patients with mid-to-high surgical risk.

### Subgroup analysis

We undertook a subgroup analysis using the fourth imputed dataset to assess the influence of potential confounders on the association between anaesthesia type and one-year postoperative mortality. The subgroup analysis indicated that the RA group consistently presented advantages across all subgroups. Although no statistically significant differences were observed in certain covariate groups, this may be due to the small sample sizes within these subgroups (Supplementary Fig. 5). We further constructed subgroup forest plots for each imputed dataset and generated a forest plot depicting the pooled effect size across the five imputation analyses (Supplementary Fig. 6).

## Discussion

This study examined the associations between anaesthesia type and one-year mortality after surgery in an elderly population. Using a large retrospective cohort design, we analysed data from consecutive elderly patients who underwent surgical procedures. Our findings indicate that patients receiving RA had a lower one-year mortality rate compared to those receiving GA (6.44% vs. 9.52%). Multivariate logistic regression analyses consistently demonstrated that RA was associated with a reduced mortality risk across various models. Specifically, the unadjusted model showed a 35% decrease in mortality odds (OR = 0.65; 95% CI: 0.58–0.75), whereas adjusted models accounting for race, sex, and comprehensive clinical variables yielded odds ratios ranging from 0.67 to 0.73 (all 95% CI below 1). These results indicate that RA is independently linked to a reduction in one-year mortality following surgery, even after controlling for potential confounders and addressing missing data.

Our findings are consistent with several previous studies, suggesting a potential benefit of RA in reducing perioperative mortality, particularly in older adults with multiple comorbidities. Anahi et al. [9] reported a significantly lower 30-day mortality rate in a RA than in a GA group (0.19% vs. 0.8%, hazard ratio 0.42, 95% confidence interval 0.21–0.83) for hip and knee replacement surgeries. In a propensity score–matched retrospective cohort analysis of 2016–2019 ACS NSQIP data, Weinstein ER et al. [33] reported significantly greater 30-day mortality with GA(OR, 1.276; 95% CI, 1.099–1.481; *P* = 0.001), whereas spinal anaesthesia significantly lowered the incidence of stroke, myocardial infarction, or death, suggesting a perioperative advantage in elderly patients with hip fractures—consistent with our findings. Luo et al. [34] Similarly, in noncardiac and nonneurosurgical procedures, the 30-day mortality rate was notably lower in patients receiving RA (0.75%) than in those receiving GA (1.31%). However, not all studies have yielded consistent findings: a randomized controlled trial by Neuman et al. [10] reported no significant effect of RA on 60-day postoperative mortality among older adults undergoing hip surgery, and a meta-analysis by Zhou et al. [35] also demonstrated no marked difference in mortality after hip fracture surgery in elderly patients. These discrepancies may result from variations in study design, sample size, patient characteristics, and surgical types. Existing trials are often limited by relatively small cohorts, and randomized clinical trials may not fully capture the causal impact of anaesthesia type on perioperative outcomes. In contrast, our study utilized a larger retrospective cohort focused on older patients, complementing previous research. The protective effect of RA may stem from multiple mechanisms, including a reduced systemic inflammatory response, avoidance of intubation and mechanical ventilation, decreased blood loss, and improved postoperative pain control [36]. A recent assessment of patients undergoing joint replacement in the United States further confirmed the benefits of neuraxial anaesthesia in lowering 30-day mortality and major complications, seemingly independent of comorbidity status, and highlighted a particularly pronounced effect in older individuals with underlying cardiopulmonary conditions [37].

RA requires fewer drugs and does not induce loss of consciousness in elderly patients, which differentiates it from GA. In older individuals, organ function often declines due to reduced drug metabolism and an increased risk of adverse reactions. Significant hypotension during induction, residual muscle relaxants, and prolonged opioid effects can lead to heightened postoperative complications, including respiratory depression, delayed awakening, postoperative delirium, and severe blood pressure fluctuations. These complications adversely affect cardiovascular and cerebral function, collectively increasing mortality rates following surgery. Therefore, anesthesiologists should prioritize RA for elderly patients during the perioperative period to enhance outcomes while effectively addressing surgical needs.

This study has several notable strengths. First, research on one-year mortality is relatively scarce, and our study specifically examines one-year mortality as the outcome, providing long-term survival data that bridge gaps in the literature. Second, our research is a real-world investigation encompassing various types of surgeries, not limited to orthopedic procedures, which significantly enhances the external validity and generalizability of the findings. Finally, we employed multiple missing data handling techniques, including multiple imputations, to ensure the robustness of our conclusions and minimize bias that could arise from missing data. These strengths confer high reliability and applicability to our study in exploring the relationship between RA and postoperative mortality, thereby offering valuable insights for clinical practice.

This study has several limitations. First, it is based on data from a single center in Singapore, which constrains both the generalizability and external validity of the findings. Thus, future multicenter investigations are warranted for validation. Second, the study population is predominantly Asian, necessitating caution when these results are applied to other racial groups. Third, as an observational study, it can detect associations between regional anaesthesia and only one-year postoperative mortality without establishing a causal relationship. Additionally, it adjusts only for measurable confounders, leaving potential unmeasured factors unaccounted for. Furthermore, the proportion of RA converted to GA and the use of combined anaesthesia remain unrecorded, which precludes a detailed assessment of these scenarios [38]. A significant limitation of this study is the lack of detailed surgical procedure categories, which may introduce indication bias. Adjustments were made for surgical risk and priority; however, the potential for unmeasured procedure-specific confounding remains. This limitation underscores the need for caution when interpreting the findings. A final limitation arises from the retrospective design of this study, which may introduce inherent biases and unmeasured confounding factors, such as the lack of data on neurocognitive impairment, which may lead patients to preferentially choose GA. Additionally, the absence of essential secondary outcome indicators, such as length of stay, complication rates, and ICU admission rates, further weakens the stability of our conclusions. Although we applied multiple analytic methods to adjust for measured covariates, prospective and multicenter trials with larger sample sizes are necessary to strengthen and validate the generalizability of the findings of this study.

In conclusion, this study in elderly patients indicated that RA may be linked to better one-year survival. Nevertheless, given the retrospective nature of this analysis, further large-scale, multicenter prospective studies are essential to elucidate the long-term effects of RA on outcomes in this population.

## Supplementary Information

Supplementary Material 1.

## Data Availability

As this study is a secondary analysis, the data are available from the Dryad Digital Repository at 10.1371/journal.pone.0182543 and are accessible to anyone who wishes to obtain them.

## Abbreviations

AIC: Akaike Information Criterion
ASA-PS: American Society of Anesthesiologists Physical Status
ATC: Average Treatment Effect for the Control
ATE: Average Treatment Effect
ATT: Average Treatment Effect for Treated
BIC: Bayesian Information Criterion
CHF: Congestive Heart Failure
CKD: Chronic Kidney Disease
CVA: Cerebrovascular Accident
DMI: Diabetes Mellitus requiring insulin therapy
E value: A metric assessing the minimum strength of association an unmeasured confounder must have with both exposure and outcome to fully explain away an observed association
GA: General Anaesthesia
IHD: Ischemic Heart Disease
IPTW: Inverse Probability of Treatment Weighting
KM: Kaplan–Meier (commonly used survival analysis method)
OR: Odds Ratio
RA: Regional Anaesthesia
RDW: Red Cell Distribution Width
SCM: Sunrise Clinical Manager
SMD: Standardized Mean Difference
STROBE: Strengthening the Reporting of Observational Studies in Epidemiology
