# Supplementary material for: Association of anaesthesia type with one-year mortality after surgery in elderly patients: a secondary retrospective cohort study
Source: BMC Anesthesiol. 2025 Jul 1;25:316. doi: 10.1186/s12871-025-03191-y (PMC12211471; doi:10.1186/s12871-025-03191-y)
Supplement: Supplementary file 1 — Supplementary Material 1. [file 12871_2025_3191_MOESM1_ESM.docx]

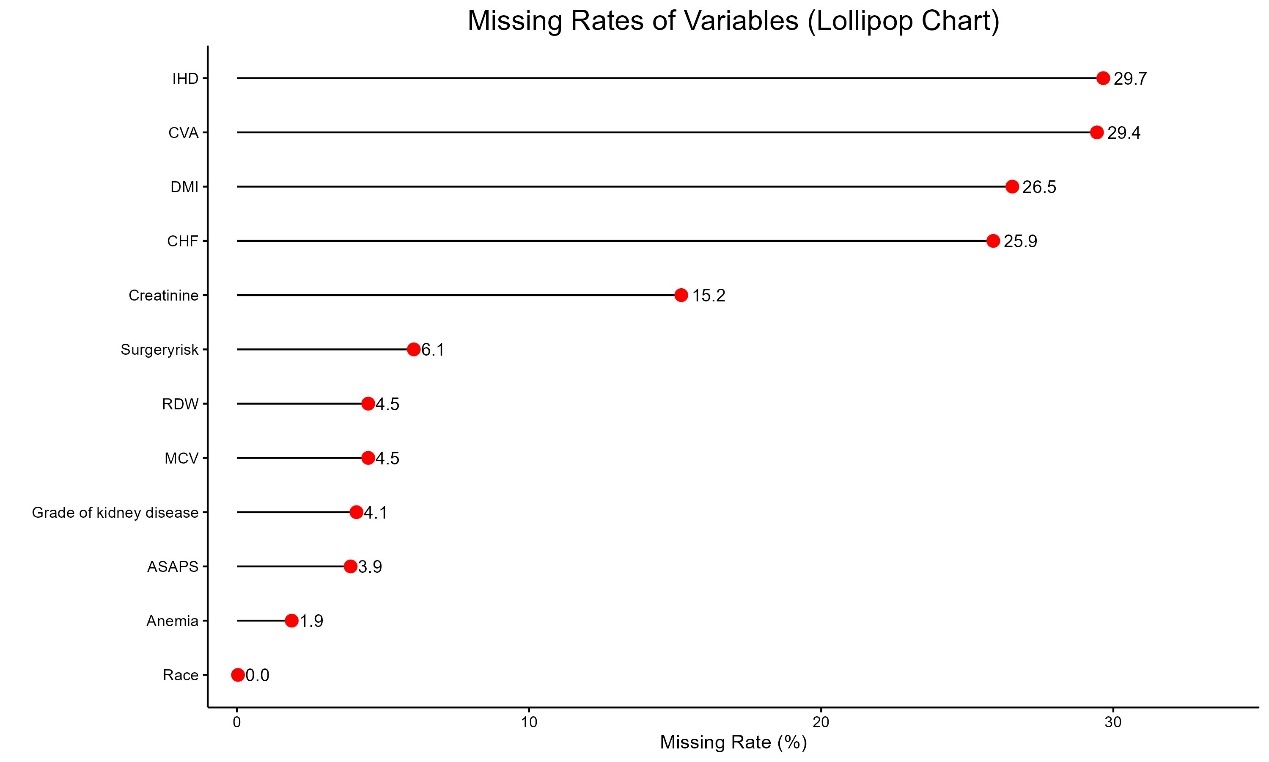


**FIGURE 3** Missing Rates of Variables (Lollipop Chart)

The lollipop chart displays the missing rates of various clinical variables, revealing a significant variability across them. The highest missing rates are observed in Ischemic Heart Disease (29.7%), Cerebrovascular Accident (29.4%), Insulin-Dependent Diabetes Mellitus (26.5%), and Congestive Heart Failure (25.9%). Moderate missing rates are noted for Surgery Risk (6.1%) and Red Cell Distribution Width (4.5%), while Anemia (1.9%) has the lowest rates. These findings highlight the need for strategies to address missing data, particularly for critical variables, to ensure the validity of subsequent analyses.


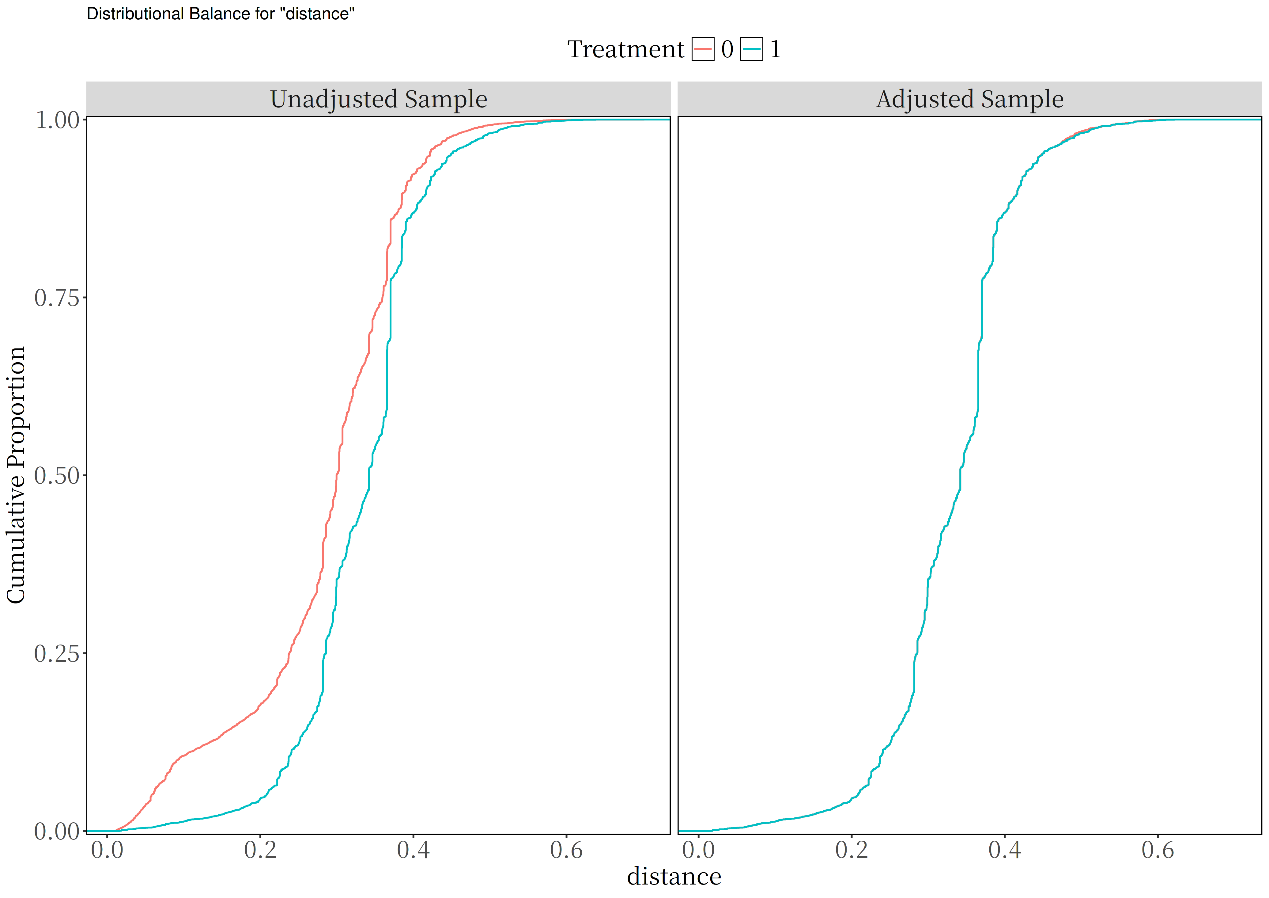


**FIGURE 4** Cumulative Distribution of "Distance" in Unadjusted vs. Adjusted Samples by Treatment Group (0 for GA Group, 1 for RA Group)


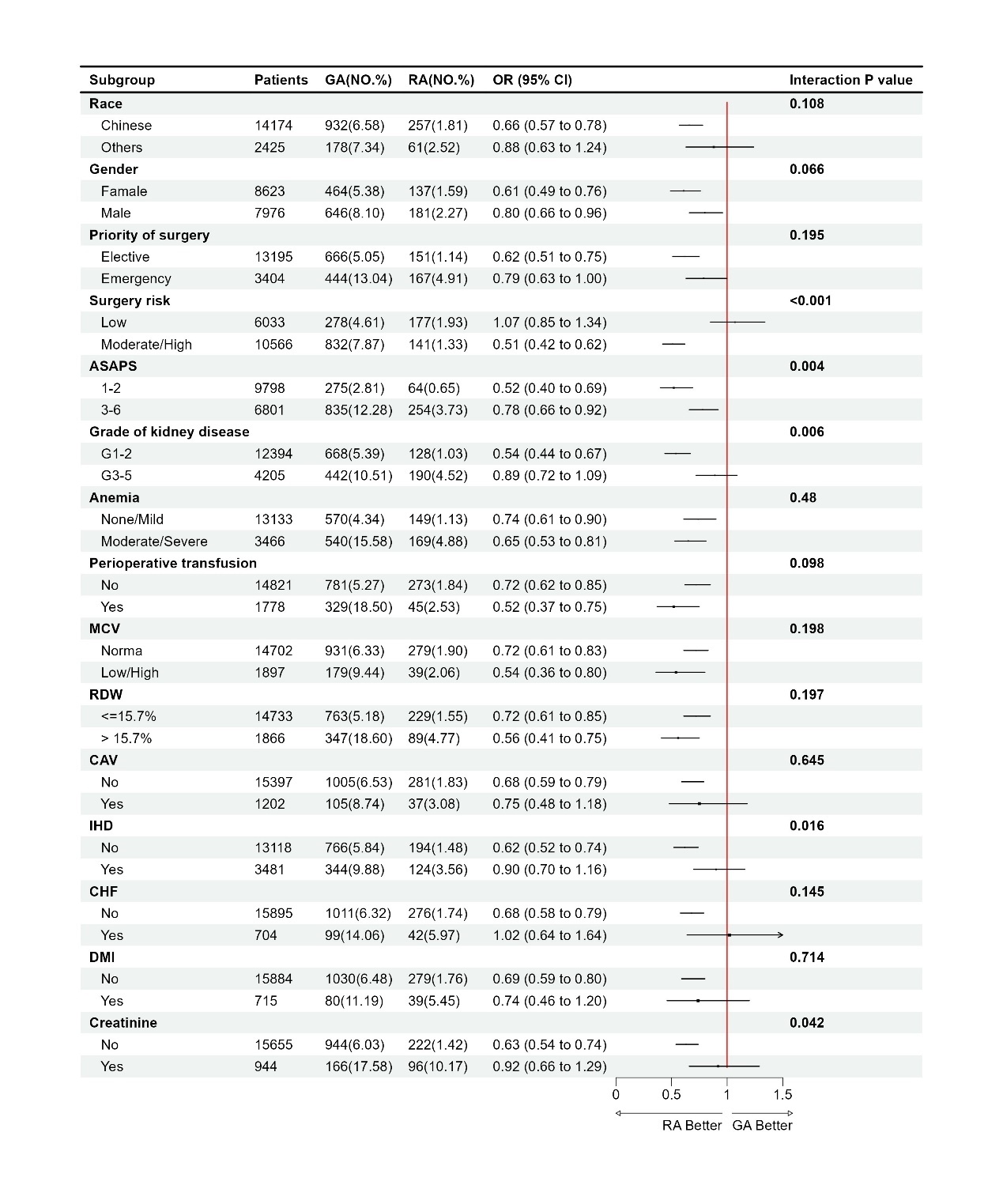


**FIGURE 5** Forest plot of the main perioperative outcomes comparing patients receiving GA versus RA across different subgroups.


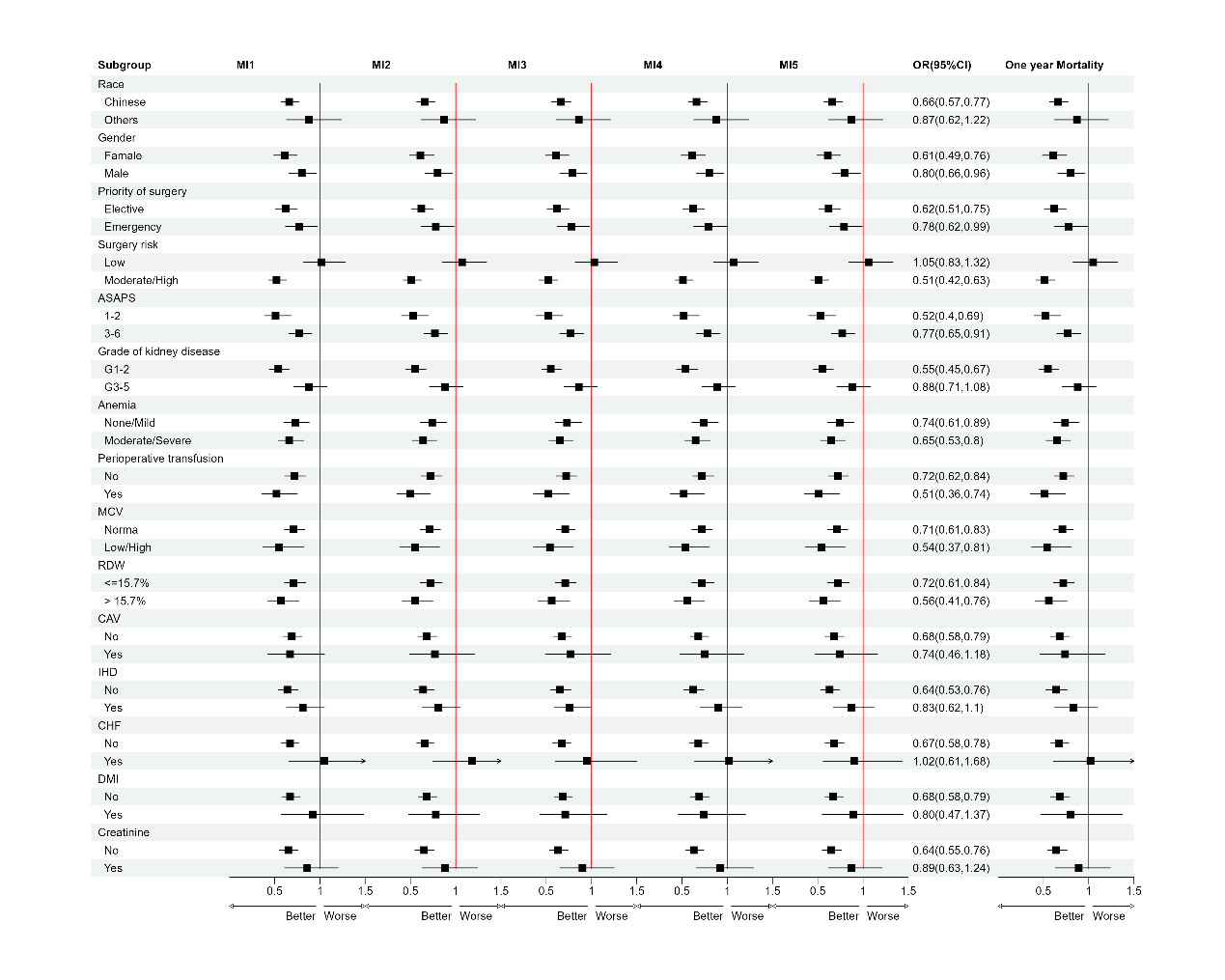


**FIGURE 6** Forest Plot of One-Year Mortality in Various Patient Subgroups, Comparing Multiple Logistic Regression Models (MI1–MI5, where MI represents Multiple Imputation Datasets).

**Table 3 | Baseline characteristics before and after propensity score matching.**

| Variable | Before PSM | | | | After PSM | | | |
| --- | --- | --- | --- | --- | --- | --- | --- | --- |
|  | Total | GA | RA | SMD | Total | GA | RA | SMD |
|  | n=16599 | n=11664 | n=4935 |  | n=9868 | n=4934 | n=4934 |  |
| Gender, n (%) |  |  |  |  |  |  |  |  |
| Female | 8623 (51.95) | 5853 (50.18) | 2770 (56.13) | 0.120 | 5525 (55.99) | 2756 (55.86) | 2769 (56.12) | 0.005 |
| Male | 7976 (48.05) | 5811 (49.82) | 2165 (43.87) | -0.120 | 4343 (44.01) | 2178 (44.14) | 2165 (43.88) | -0.005 |
| Race, n (%) |  |  |  |  |  |  |  |  |
| Chinese | 14174 (85.39) | 9882 (84.72) | 4292 (86.97) | 0.067 | 8612 (87.27) | 4321 (87.58) | 4291 (86.97) | -0.018 |
| Indian | 764 (4.6) | 543(4.66) | 221(4.48) | -0.009 | 414 (4.2) | 193(3.91) | 221(4.48) | 0.027 |
| Malay | 838(5.05) | 582 (4.99) | 256(5.19) | 0.009 | 510(5.17) | 254(5.15) | 256(5.19) | 0.002 |
| Others | 823 (4.96) | 657 (5.63) | 166 (3.36) | -0.126 | 332(3.36) | 166(3.36) | 166(3.36) | 0.000 |
| Grade of kidney disease, n (%) |  |  |  |  |  |  |  |  |
| G1 | 5199 (31.32) | 3674 (31.50) | 1525 (30.90) | -0.013 | 3081 (31.22) | 1556 (31.54) | 1525 (30.91) | -0.014 |
| G3 | 3076 (18.53) | 2172 (18.62) | 904 (18.32) | -0.008 | 1796 (18.2) | 892 (18.08) | 904 (18.32) | 0.006 |
| G2 | 7195 (43.35) | 5108 (43.79) | 2087 (42.29) | -0.03 | 4207 (42.63) | 2120 (42.97) | 2087 (42.30) | -0.014 |
| G4 and 5 | 1129 (6.8) | 710 (6.09) | 419 (8.49) | 0.086 | 784 (7.94) | 366 (7.42) | 418 (8.47) | 0.038 |
| RDW, n (%) |  |  |  |  |  |  |  |  |
| <=5.7% | 14733 (88.76) | 10248 (87.86) | 4485 (90.88) | 0.105 | 8999 (91.19) | 4514 (91.49) | 4485 (90.90) | -0.02 |
| > 15.7% | 1866 (11.24) | 1416 (12.14) | 450 (9.12) | -0.105 | 869 (8.81) | 420 (8.51) | 449 (9.10) | 0.02 |
| MCV, n (%) |  |  |  |  |  |  |  |  |
| High | 533 (3.21) | 377 (3.23) | 156 (3.16) | -0.004 | 313 (3.17) | 157 (3.18) | 156 (3.16) | -0.001 |
| Low | 1364 (8.22) | 1025 (8.79) | 339 (6.87) | -0.076 | 669 (6.78) | 330 (6.69) | 339 (6.87) | 0.007 |
| Norma | 14702 (88.57) | 10262 (87.98) | 4440 (89.97) | 0.066 | 8886 (90.05) | 4447 (90.13) | 4439 (89.97) | -0.005 |
| Anemia, n (%) |  |  |  |  |  |  |  |  |
| Mild | 3986 (24.01) | 2811 (24.10) | 1175 (23.81) | -0.007 | 2367 (23.99) | 1192 (24.16) | 1175 (23.81) | -0.008 |
| Moderate | 3360 (20.24) | 2348 (20.13) | 1012 (20.51) | 0.009 | 1941 (19.67) | 930 (18.85) | 1011 (20.49) | 0.041 |
| None | 9147 (55.11) | 6415 (55.00) | 2732 (55.36) | 0.007 | 5529 (56.03) | 2797 (56.69) | 2732 (55.37) | -0.027 |
| Severe | 106 (0.64) | 90 (0.77) | 16 (0.32) | -0.079 | 31 (0.31) | 15 (0.30) | 16 (0.32) | 0.004 |
| CVA, n (%) |  |  |  |  |  |  |  |  |
| No | 15397 (92.76) | 10814 (92.71) | 4583 (92.87) | 0.006 | 9187 (93.1) | 4605 (93.33) | 4582 (92.87) | -0.018 |
| Yes | 1202 (7.24) | 850 (7.29) | 352 (7.13) | -0.006 | 681 (6.9) | 329 (6.67) | 352 (7.13) | 0.018 |
| CHF, n (%) |  |  |  |  |  |  |  |  |
| No | 15895 (95.76) | 11161 (95.69) | 4734 (95.93) | 0.012 | 9493 (96.2) | 4760 (96.47) | 4733 (95.93) | -0.028 |
| Yes | 704 (4.24) | 503 (4.31) | 201 (4.07) | -0.012 | 375 (3.8) | 174 (3.53) | 201 (4.07) | 0.028 |
| DMI, n (%) |  |  |  |  |  |  |  |  |
| No | 15884 (95.69) | 11203 (96.05) | 4681 (94.85) | -0.054 | 9394 (95.2) | 4713 (95.52) | 4681 (94.87) | -0.029 |
| Yes | 715 (4.31) | 461 (3.95) | 254 (5.15) | 0.054 | 474 (4.8) | 221 (4.48) | 253 (5.13) | 0.029 |
| Creatinine, n (%) |  |  |  |  |  |  |  |  |
| No | 15655 (94.31) | 11063 (94.85) | 4592 (93.05) | -0.071 | 9226 (93.49) | 4635 (93.94) | 4591 (93.05) | -0.035 |
| Yes | 944 (5.69) | 601 (5.15) | 343 (6.95) | 0.071 | 642 (6.51) | 299 (6.06) | 343 (6.95) | 0.035 |
| Perioperative transfusion, n (%) |  |  |  |  |  |  |  |  |
| 0 unit | 14821 (89.29) | 10207 (87.51) | 4614 (93.50) | 0.243 | 9228 (93.51) | 4615 (93.53) | 4613 (93.49) | -0.002 |
| 1 unit | 1427 (8.6) | 1166 (10.00) | 261 (5.29) | -0.21 | 517 (5.24) | 256 (5.19) | 261 (5.29) | 0.005 |
| 2 or more units | 351 (2.11) | 291 (2.49) | 60 (1.22) | -0.117 | 123 (1.25) | 63 (1.28) | 60 (1.22) | -0.006 |
| Surgery Risk, n (%) |  |  |  |  |  |  |  |  |
| High | 1258 (7.58) | 1181 (10.13) | 77 (1.56) | -0.691 | 146 (1.48) | 69 (1.40) | 77 (1.56) | 0.013 |
| Low | 6033 (36.35) | 4283 (36.72) | 1750 (35.46) | -0.026 | 3394 (34.39) | 1644 (33.32) | 1750 (35.47) | 0.045 |
| Moderate | 9308 (56.08) | 6200 (53.16) | 3108 (62.98) | 0.203 | 6328 (64.13) | 3221 (65.28) | 3107 (62.97) | -0.048 |
| Priority of Surgery, n (%) |  |  |  |  |  |  |  |  |
| Elective | 13195 (79.49) | 9314 (79.85) | 3881 (78.64) | -0.03 | 7866 (79.71) | 3985 (80.77) | 3881 (78.66) | -0.051 |
| Emergency | 3404 (20.51) | 2350 (20.15) | 1054 (21.36) | 0.03 | 2002 (20.29) | 949 (19.23) | 1053 (21.34) | 0.051 |
| ASAPS, n (%) |  |  |  |  |  |  |  |  |
| Ⅰ | 411 (2.48) | 280 (2.40) | 131 (2.65) | 0.016 | 274 (2.78) | 143 (2.90) | 131 (2.66) | -0.015 |
| Ⅱ | 9387 (56.55) | 6326 (54.24) | 3061 (62.03) | 0.161 | 6196 (62.79) | 3136 (63.56) | 3060 (62.02) | -0.032 |
| Ⅲ | 6090 (36.69) | 4429 (37.97) | 1661 (33.66) | -0.091 | 3237 (32.8) | 1576 (31.94) | 1661 (33.66) | 0.036 |
| Ⅳ-Ⅵ | 711 (4.28) | 629 (5.39) | 82 (1.66) | -0.292 | 161 (1.63) | 79 (1.60) | 82 (1.66) | 0.005 |

The fourth imputed dataset selected based on the lowest Akaike Information Criterion (AIC) and Bayesian Information Criterion (BIC) values.

**Table 4** **| Associations between anesthesia type and one-year postoperative mortality of elderly surgical patients in the three propensity-score methods analyses**

| Logistic regression model | Adjusted variables | No. | OR (95% CI) P value |
| --- | --- | --- | --- |
| Propensity score matching | Adjusted II | 9868 | 0.76(0.65, 0.88) 0.001 |
| Propensity score adjustment | Adjusted II + Propensity score | 16599 | 0.74 (0.64, 0.86) <0.001 |
| IPTW | Adjusted II | 16599 |  |
| ATE |  |  | 0.71 (0.60, 0.84) <0.001 |
| ATT |  |  | 0.72 (0.62, 0.83) <0.001 |
| ATC |  |  | 0.71 (0.59, 0.86) <0.001 |

OR, Odds Ratio; CI, conﬁdence interval; IPTW, inverse-probability-of-treatment weighted; ATT average treatment effect for treated; ATC average treatment effect for control; ATE average treatment effect for all. Adjusted II: Adjusted for Race; Gender. Adjust II model adjust for: Race ; Gender; ASAPS ; CVA ; IHD ; CHF ; DMI ; Creatinine ; Surgery risk ; Anemia ; Grade of kidney disease ; Priority of surgery; Perioperative transfusion; MCV ; RDW

**Table 5| Associations between the anesthesia type and the endpoint of mortality at different time intervals**

| Time(days) | Mortality Proportion  [NO. (%)] | |  | Multivariable analysis - Odds Ratio (95% CI) P value | | |
| --- | --- | --- | --- | --- | --- | --- |
|  | GA | RA |  | Non-adjusted | Adjust I | Adjust II |
| 30 | 291 (2.49) | 62 (1.26) |  | 0.50 (0.38, 0.66) <0.0001 | 0.51 (0.39, 0.67) <0.0001 | 0.73 (0.49, 1.09) 0.1236 |
| 60 | 421 (3.61) | 107 (2.17) |  | 0.59 (0.48, 0.73) <0.0001 | 0.60 (0.49, 0.75) <0.0001 | 0.76 (0.56, 1.04) 0.0873 |
| 90 | 516 (4.42) | 135 (2.74) |  | 0.61 (0.50, 0.74) <0.0001 | 0.62 (0.51, 0.75) <0.0001 | 0.74 (0.57, 0.98) 0.0337 |
| 180 | 765 (6.56) | 202 (4.09) |  | 0.61 (0.52, 0.71) <0.0001 | 0.62 (0.53, 0.73) <0.0001 | 0.68 (0.54, 0.85) 0.0009 |
| 360 | 1110 (9.52) | 318 (6.44) |  | 0.65 (0.58, 0.75) <0.0001 | 0.67 (0.59, 0.76) <0.0001 | 0.73 (0.61, 0.88) 0.0009 |

Non-adjusted model adjust for: None. Adjust I model adjust for: Race; Gender. Adjust II model adjust for: Race ; Gender; ASAPS ; CVA ; IHD ; CHF ; DMI ; Creatinine ; Surgery risk ; Anemia ; Grade of kidney disease ; Priority of surgery; Perioperative transfusion; MCV ; RDW . DV-Imp: Dataset with missing values handled using dummy variable adjustments. MI : Multiple imputation datasets.
